# The global battle against SARS-CoV-2 and COVID-19

**DOI:** 10.7150/ijbs.45587

**Published:** 2020-03-15

**Authors:** Chu-Xia Deng

**Affiliations:** 1 Editor in Chief, International Journal of Biological Sciences; 2 Chair Professor, Faculty of Health Sciences, University of Macau, Macau SAR, China

On December 31, China announced “it is probing a mystery viral pneumonia outbreak in Wuhan”. Since then, the virus, known as Severe Acute Respiratory Syndrome Coronavirus 2 (SARS-CoV-2)” is threatening the life of people of at least 140 countries including China (Table 1). On February 11, the World Health Organization (WHO) named the disease caused by SARS-CoV-2 as Coronavirus Disease-19 (COVID-19). COVID-19 patients exhibit flu-like symptoms, such as persistent coughing, fever, shortness of breath, and difficulty breathing, which are similar to the Severe Acute Respiratory Syndrome (SARS), and the Middle East Respiratory Syndrome (MERS).

Because SARS-CoV-2 is a new pathogen, people of all ages have no immunity to it and generally susceptible to infection. Just in about 2.5 months, COVID-19 has been spreading quickly to over 140 countries, infected more than 156,000 people, ranging from newborn to 98 years of age, and killed at least 5,800 patients, mostly in Wuhan, Hubei province of China. Compared with its close related coronavirus family member SARS-CoV and MERS-CoV, which infected 8096 and 2494 people in year 2003 and year 2012, respectively, the outbreak of COVID-19 is much more serious with its high virulence. Although SARS-CoV-2 exhibits a lower fatality rate compared to SARS-CoV and MERS-CoV, the virus has already killed at least 1.8 times more people than SARS-CoV and MERS-CoV combined together (Table 1).

Up to today, China has taken unprecedented public health measures with centralized national medical support, and has achieved significant results in slowing down the spread of the epidemic and blocking the sources of transmission, preventing hundreds of thousands of new cases of pneumonia. While the rate of infection comes down gradually in China, the scales of infection in some other countries are steadily rising. The war against COVID-19 will continue on a global scale. Thus, the knowledge the virus, the mechanism of its infection, the characteristics of the epidemic transmission are essentially important for effective fighting against this deadly disease.

In this special issue, we have 11 reports, which share our understanding of SARS-CoV-2 and COVID-19 from different angles. Zheng indicated that SARS-CoV-2 is an emerging new coronavirus that causes a global threat, and summarized the key events occurred during the early outbreak, the basic characteristics of the pathogen, the signs and symptoms of the infected patients, the possible transmission pathways of the virus, the understanding on the origin and evolution of the virus, as well as the chemotherapeutic options under development 1. Ye et al. overviewed the existing knowledge about 7 human coronavirus (HCoVs), with a focus on the history of their discovery as well as their zoonotic origins and interspecies transmission. They also compared and contrasted the different HCoVs from a perspective of virus evolution and genome recombination 2. Lo et al. reported their experience on the evaluation of SARS-CoV-2 RNA shedding in clinical specimens and clinical features of all 10 patients in Macau, and recommended the assessment of both fecal and respiratory specimens for enhancing diagnostic sensitivity 3.

Because of no specific anti-virus drugs or vaccines are available for the treatment of this sudden and lethal disease, many drugs have been used in the therapy. Yang et al. reported that greater than 85% of COVID-19 patients in China have been receiving Traditional Chinese Medicine (TCM) treatment, and presented the clinical evidence showing the beneficial effect of TCM in the treatment of the patients 4. Zhou and Zhao pointed out the great importance of using therapeutic neutralizing antibodies (NAbs) to control the spread and re-emergence of SARS-CoV-2 and assert that the development of NAbs therefore should be a high priority in near future 5. Yang and Shen discussed the implication of the endocytic pathway and autophagy in viral infection and how novel therapeutic approaches could be developed by targeting these processes for treatment of COVID-19 6.

No one was prepared for this highly infectious and swiftly transmitting disease, creating unprecedented strain on healthcare providers and the overall system. Indeed, the rapid transmission of the COVID-19 has mounted serious challenges to both the patients and the health workers stationed at the epicenter of the outbreak 7. Xiang et al. brought up an issue of high infection rate among health workers in China, mainly due to the lack of experience in handling it in the early stages of the epidemic 8. On the other hand, more than 300 Chinese patients with psychiatric disorders have been with the SARS-CoV-2 since the outbreak of COVID-19 9. Patients, health professionals and the general public are under insurmountable psychological pressure, which may lead to various mental health problems, such as anxiety, fear, depression, and insomnia. Emergency psychological teams have been urgently established at regional and national levels and provided mental health services for those in need as part of the overall deployment of the disease control 7,9.

Understanding the knowledge, attitudes and behaviors of residents towards COVID-19 during the early stage of disease outbreak could help the authority effectively implement preventive and control measures. Zhong et al. conducted an epidemiological survey involving 6,910 residents in China, and the results showed that even during the rapid rise period of the COVID-19 outbreak, most residents had good knowledge of COVID-19 and were optimistic about the epidemic control. To reduce the infection risk, they acted with caution in daily life, and took appropriate preventive measures as recommended by the health authorities 10 . Finally, we bring up a comprehensive review on what we have learned and to be learned by Li et al, who cover the basics about the epidemiology, etiology, virology, diagnosis, treatment, prognosis, and prevention of the disease in comparison with SARS and MERS 11.

As the fight to COVID-19 continues, we hope that this special issue will help researchers from various fields to have a better understanding of this dangerous virus and join the urgent fight against this deadly disease. We believe the information provided in this special issue should facilitate the fighting against SARS-CoV-2 and the related COVID-19, a global war that we must win and we will win although with huge expenses, the extent of which remains unclear.

## Figures and Tables

**Table 1:** Comparison among COVID-19, SARS and MERS.

Name of Disease (Name of Virus)	Coronavirus Disease-2019 (SARS-CoV-2) *	Severe Acute Respiratory Syndrome (SARS-CoV)	Middle East Respiratory Syndrome (MERS-CoV)
Major symptoms	Fever, cough, shortness of breath, and difficult to breath, pneumonia	Fever, cough, sore throat, muscle pain, lethargy, headache, diarrhea, shivering, and breathing difficulties	fever, cough, shortness of breath, muscle pain, vomiting, and abdominal pain
Total cases (Death)	>156,000 (>5800)	8096 (774)	2499 (861)
Fatality rate	About 3.7%	9.6%	34.4%
Countries affected	>140	29	26
Likely animal hosts	Bats, pangolin	Bats, civet cats	Dromedary camels

* Updated by March 15, 2020.

## References

[1] Zheng J (2020). SARS-CoV-2: an Emerging Coronavirus that Causes a Global Threat. Int J Biol Sci.

[2] Ye ZW, Yuan S, Yuen KS, Fung SY, Chan CP, Jin DY (2020). Zoonotic origins of human coronaviruses. Int J Biol Sci.

[3] Lo IL, Lio CF, Cheong HH, Lei CI, Cheong TH, Zhong X, Tian Y, Sin NN (2020). Evaluation of SARS-CoV-2 RNA shedding in clinical specimens and clinical characteristics of 10 patients with COVID-19 in Macau. Int J Biol Sci.

[4] Yang Y, Islam MS, Wang J, Li Y, Chen X (2020). Traditional Chinese Medicine in the Treatment of Patients Infected with 2019-New Coronavirus (SARS-CoV-2): A Review and Perspective. Int J Biol Sci.

[5] Zhou G, Zhao Q (2020). Perspectives on therapeutic neutralizing antibodies against the Novel Coronavirus SARS-CoV-2. Int J Biol Sci.

[6] Yang N, Shen HM (2020). Targeting the Endocytic Pathway and Autophagy Process as a Novel Therapeutic Strategy in COVID-19. Int J Biol Sci.

[7] Li W, Yang Y, Liu ZH, Zhao YJ, Zhang Q, Zhang L, Cheung T, Xiang YT (2020). Progression of Mental Health Services during the COVID-19 Outbreak in China. Int J Biol Sci.

[8] Xiang YT, Jin Y, Wang Y, Zhang Q, Zhang L, Cheung T (2020). Tribute to health workers in China: A group of respectable population during the outbreak of the COVID-19. Int J Biol Sci.

[9] Xiang YT, Zhao YJ, Liu ZH, Li XH, Zhao N, Cheung T, Ng CH (2020). The COVID-19 outbreak and psychiatric hospitals in China: managing challenges through mental health service reform. Int J Biol Sci.

[10] Zhong BL, Luo W, Li HM, Zhang QQ, Liu XG, Li WT, Li Y (2020). Knowledge, attitudes, and practices towards COVID-19 among Chinese residents during the rapid rise period of the COVID-19 outbreak: a quick online cross-sectional survey. Int J Biol Sci.

[11] Yi Y, Lagniton PNP, Ye S, Li E, Xu RH (2020). COVID-19: what has been learned and to be learned about the novel coronavirus disease. Int J Biol Sci.

